# Improving Voice Spoofing Detection Through Extensive Analysis of Multicepstral Feature Reduction †

**DOI:** 10.3390/s25154821

**Published:** 2025-08-05

**Authors:** Leonardo Mendes de Souza, Rodrigo Capobianco Guido, Rodrigo Colnago Contreras, Monique Simplicio Viana, Marcelo Adriano dos Santos Bongarti

**Affiliations:** 1 Department of Computer Science and Statistics, Institute of Biosciences, Letters and Exact Sciences, São Paulo State University, São José do Rio Preto 15054-000, SP, Brazil; leonardo.m.souza@unesp.br (L.M.d.S.); guido@ieee.org (R.C.G.); 2 Department of Science and Technology, Federal University of São Paulo, São José dos Campos 12247-014, SP, Brazil; 3 Department of Computing, Federal University of São Carlos, São Carlos 13565-905, SP, Brazil; moniquesimplicioviana@estudante.ufscar.br; 4 Weierstraß Institute for Applied Analysis and Stochastics, 10117 Berlin, Germany; bongarti@wias-berlin.de

**Keywords:** spoofing detection, dimensionality reduction, pattern recognition, cepstral analysis, machine learning

## Abstract

Voice biometric systems play a critical role in numerous security applications, including electronic device authentication, banking transaction verification, and confidential communications. Despite their widespread utility, these systems are increasingly targeted by sophisticated spoofing attacks that leverage advanced artificial intelligence techniques to generate realistic synthetic speech. Addressing the vulnerabilities inherent to voice-based authentication systems has thus become both urgent and essential. This study proposes a novel experimental analysis that extensively explores various dimensionality reduction strategies in conjunction with supervised machine learning models to effectively identify spoofed voice signals. Our framework involves extracting multicepstral features followed by the application of diverse dimensionality reduction methods, such as Principal Component Analysis (PCA), Truncated Singular Value Decomposition (SVD), statistical feature selection (ANOVA F-value, Mutual Information), Recursive Feature Elimination (RFE), regularization-based LASSO selection, Random Forest feature importance, and Permutation Importance techniques. Empirical evaluation using the ASVSpoof 2017 v2.0 dataset measures the classification performance with the Equal Error Rate (EER) metric, achieving values of approximately 10%. Our comparative analysis demonstrates significant performance gains when dimensionality reduction methods are applied, underscoring their value in enhancing the security and effectiveness of voice biometric verification systems against emerging spoofing threats.

## 1. Introduction

Voice authentication systems have increasingly become integral for secure user identification due to their practicality, uniqueness, and non-invasiveness, leveraging the inherent characteristics of individual vocal traits [1,2,3]. Despite these advantages, voice-based authentication remains vulnerable to sophisticated spoofing attacks, which involve the manipulation or artificial synthesis of voice signals using advanced techniques, particularly those based on artificial intelligence [4,5]. Such spoofing methods significantly threaten the reliability and security of biometric voice authentication systems [6].

Among the most successful feature categories for detecting spoofed voice signals are cepstral features, particularly Mel-Frequency Cepstral Coefficients (MFCCs) and their variants [7]. These features are capable of capturing fine-grained spectral details of speech, making them highly effective in distinguishing genuine speech from synthetic or manipulated signals. More recently, multicepstral approaches—based on the aggregation or fusion of multiple cepstral representations—have emerged as a powerful strategy to further improve performance [8]. These approaches offer complementary perspectives of the signal through diverse projection and mapping strategies. However, the enhanced representational richness of multicepstral features comes at a cost: very high dimensionality. This not only leads to increased memory and computational demands but also introduces challenges, such as overfitting, sparse data representations, and degraded classifier performance—a phenomenon commonly referred to as the curse of dimensionality [9,10,11]. To address this, dimensionality reduction becomes a crucial preprocessing step, enabling the condensation of the most informative components of the feature space while minimizing redundancy and computational burden.

This article is an extended version of a preliminary study [12] in which we initiated the investigation of dimensionality reduction techniques applied to multicepstral features in the context of voice spoofing detection. While the earlier version focused on a narrower set of methods, this work expands upon that foundation in several important ways. First, we introduce a dedicated section reviewing related works to better contextualize the contributions. Second, we significantly broaden the scope of the experimental framework by including additional dimensionality reduction strategies. These now encompass techniques such as Principal Component Analysis (PCA), Truncated Singular Value Decomposition (SVD) [13], ANOVA F-value selection, Mutual Information selection, Recursive Feature Elimination (RFE), LASSO regression-based selection, Random Forest-based importance selection, and Permutation Importance analysis. Finally, we also incorporate noncepstral features into the analysis, further enriching the representation of speech signals and allowing for a more comprehensive evaluation of discriminative capabilities.

The main scientific contributions of this extended work can be summarized as follows:

- The incorporation of noncepstral features to enrich the descriptive power of the voice signal representation;
- A broader experimental analysis comprising additional dimensionality reduction techniques and a more diverse set of configurations;
- Extensive validation on the ASVSpoof 2017 v2.0 benchmark dataset, demonstrating the efficiency of the proposed techniques in various experimental conditions.

The structure of this paper is outlined as follows: Section 2 discusses recent advancements and the relevant literature on voice-based spoofing detection through artificial intelligence methods. Section 3 introduces a conceptual and procedural summary of cepstral coefficient extraction in a matrix format. The methodology and experimental framework adopted in this study are described in Section 4. Section 5 outlines the specific configurations and parameterizations derived from the generalized framework. The empirical results, along with detailed interpretations, are presented in Section 6. Lastly, Section 7 offers final reflections and proposes future research avenues.

## 2. Related Works

Voice authentication systems (VASs) typically operate in two main phases: enrollment and verification. During enrollment, speaker-specific features are extracted to train a classification model. In the verification phase, users submit real-time voice samples, which are compared against previously stored models [14]. However, these systems are particularly vulnerable to spoofing attacks, especially during signal capture or transmission. Replay attacks—where a previously recorded voice is played back to the system—are among the most prevalent and effective methods of deception. Other forms, including synthetic or converted voices generated via tools such as Skype Voice Changer, further complicate detection.

To promote innovation in anti-spoofing strategies, the ASVspoof Challenge was launched in 2015 [15], followed by subsequent editions in 2017, 2019, and 2021 [16,17,18]. These initiatives provided benchmark datasets containing genuine and spoofed utterances, supporting the development of standardized countermeasures. In the 2015 edition, Patel and Patil [19] proposed a Gaussian Mixture Model (GMM) fed by features derived from Cochlear Filter Cepstral Coefficients (CFCCs) combined with instantaneous frequency (IF). Their approach mimicked auditory processing mechanisms and achieved remarkable performance, with EERs of 0.4079% for known attacks and 2.0132% for unknown types. Sahidullah et al. [20] systematically compared 19 feature sets grouped into short-term spectral, phase-based, and long-term descriptors. Their work combined these with GMM and Support Vector Machine (SVM) classifiers, achieving EERs as low as 0.07% in matched conditions and 1.67% in mismatched scenarios.

The Constant-Q Transform (CQT), a method initially applied to music signal processing, was adapted for spoofing detection by Todisco et al. [21]. Offering better resolution in low and high-frequency ranges, CQT-based features achieved EERs of 0.048% for known attacks. Yang and Das [22] improved upon this by introducing frame-based low-frequency normalization, which enhanced robustness to environmental variability. Their model, using SVMs and ResNet architectures, reached an EER of 10.31% on ASVspoof 2017. Traditional descriptors, such as identity vectors (i-vectors), have also shown promise. Hanilçi [23] demonstrated that combining i-vectors with GMMs halved the EER relative to using GMMs alone, validating their effectiveness for voice-based spoofing detection.

Deep learning methodologies have gained traction for their capacity to model complex patterns. Qian et al. [24] explored five neural architectures—including Autoencoders, basic DNNs, and LSTM-based RNNs—achieving EERs near 0% for known spoofing conditions. Huang and Pun [25] introduced a hybrid DenseNet-LSTM model using CQCC and MFCC features, reducing EER to 7.34% on ASVspoof 2017. Gomez-Alanis et al. [26] tackled noise robustness with a Gated Recurrent Convolutional Neural Network (GRCNN), outperforming existing models across three ASVspoof datasets. More recently, Himawan et al. [27] addressed overfitting challenges by leveraging transfer learning. They used AlexNet, a CNN pre-trained on over one million images, to extract spectrogram embeddings, which were then classified via SVMs. This approach produced new benchmarks and achieved an EER of 11.21%, outperforming several state-of-the-art methods.

Our previous studies have laid a strong foundation for exploring multicepstral representations in voice-based biometric systems. In an early investigation, we introduced mapping strategies for combining diverse cepstral coefficients, such as MFCC, CQCC, BFCC, and LFCC, into enriched vectorial representations for spoofing detection, emphasizing the potential of multi-projection techniques to enhance signal discriminability [28]. Subsequently, we extended this approach to the medical domain, where we applied multicepstral features to dysphonia detection and analyzed the effect of various projection configurations, highlighting the generalizability of these representations beyond spoofing detection [8]. Building upon this foundation, we further proposed the use of metaheuristic algorithms, such as the Genetic Algorithm (GA), Particle Swarm Optimization (PSO), and Grey Wolf Optimization (GWO), to optimize feature selection in multicepstral spaces, achieving performance gains over baseline projections [29]. Most recently, we conducted a systematic comparison of three dimensionality reduction strategies, i.e., Singular Value Decomposition (SVD), Autoencoders (AEs), and Genetic Algorithms, demonstrating their efficacy in mitigating the curse of dimensionality and improving EER by up to 7.11% on the ASVSpoof 2017 v2.0 dataset [12]. These studies collectively underscore our ongoing effort to optimize the use of multicepstral features through dimensionality reduction and metaheuristic optimization in the context of secure voice authentication.

To provide a consolidated view of the techniques discussed in this section, Table 1 presents a comparative summary of relevant studies addressing voice spoofing detection. The table highlights the main methodologies adopted, datasets employed, best-reported EERs, and key contributions of each work. This comparative synthesis underscores the evolution of approaches in the literature, from handcrafted features and traditional classifiers to deep neural networks and hybrid models. Additionally, it situates our prior investigations within this broader research landscape, illustrating our progressive exploration of multicepstral representations, dimensionality reduction, and metaheuristic optimization in voice-based biometric systems.

Despite the significant advances in spoofing detection, most studies remain centered on cepstral-based representations, particularly MFCCs, CQCCs, and their derivatives. While such descriptors have demonstrated remarkable discriminative power, they often omit complementary aspects of the signal that could be captured by noncepstral features. Furthermore, the increasing complexity of multicepstral representations, driven by fusions, projections, and transformations, has introduced high-dimensional feature spaces, which are not only computationally demanding but also prone to overfitting and sparsity issues. In this way, dimensionality reduction techniques have not received proportional attention in the literature, despite their well-known potential to mitigate the curse of dimensionality. Methods such as PCA, SVD, Mutual Information ranking, and model-driven feature selection remain underexplored within the context of voice spoofing detection. Similarly, the joint use of cepstral and noncepstral features has not been systematically investigated in this domain, leaving a gap in the understanding of how different signal descriptors interact and complement each other under reduced-dimensionality scenarios. This work aims to fill these gaps by proposing an extended experimental framework that incorporates a rich combination of cepstral and noncepstral features, systematically assessed under various dimensionality reduction strategies. By doing so, we seek to uncover more compact, discriminative, and robust representations capable of enhancing the performance and generalizability of voice authentication systems in the presence of spoofing attacks.

## 3. Cepstral Feature Extraction Fundamentals

In voice-based classification systems, a crucial initial step involves extracting meaningful descriptors from raw audio signals. These descriptors, or features, serve as compact and informative representations that facilitate pattern recognition and decision-making. In the context of audio analysis, several low-level features derived from the temporal domain, such as signal energy [31], entropy [32], zero-crossing rate (ZCR) [33], and the Teager Energy Operator (TEO) [34], are widely used due to their simplicity and computational efficiency. However, such descriptors may be insufficient when a more nuanced understanding of vocal characteristics is required, particularly in tasks involving pathological speech detection or biometric authentication under adverse conditions.

To address this, spectral and cepstral-based approaches are often preferred. These methods capture the harmonic structure and modulation properties of speech, providing richer information on both the vocal tract and excitation source. Among them, MFCCs and linear prediction cepstral coefficients (LPCCs) are particularly popular, offering high discriminative power in a variety of speech-related applications. These cepstral features are typically extracted through a multi-stage pipeline that includes the following [8,28]:

(i) A pre-emphasis filter to compensate for high-frequency attenuation;
(ii) Segmentation of the signal into short overlapping frames;
(iii) Application of window functions;
(iv) Transformation into the frequency domain, usually via Fast Fourier Transform (FFT);
(v) Mapping onto perceptual or linear filter banks. The final step involves the computation of cepstral coefficients from the resulting spectral envelopes.

Formally, a cepstral extraction method Φ is applied to an input signal $x\in R^{n}$, producing a matrix $C_{x}^{\Phi }\in R^{n_{\mathrm{ceps}}\times n_{\mathrm{frames}}}$, where $n_{\mathrm{ceps}}$ is the number of cepstral coefficients computed per frame and $n_{\mathrm{frames}}$ is the total number of frames considered. This matrix captures the dynamic evolution of cepstral features throughout the signal’s duration:

(1) $$C_{x}^{\Phi }=\left[\begin{array}{ccc} — & {\vec{c}}_{1} & —\\ — & {\vec{c}}_{2} & —\\ \; & \vdots & \\ — & {\vec{c}}_{n_{\mathrm{ceps}}} & — \end{array}\right]\in R^{n_{\mathrm{ceps}}\times n_{\mathrm{frames}}},$$

where each column vector ${\vec{c}}_{i}\in R^{n_{\mathrm{ceps}}}$ corresponds to the set of cepstral features extracted from the *i*th frame of *x*. Additional details on the mathematical formulation and algorithmic implementation of these cepstral techniques can be found in Prabakaran and Shyamala [35] and Alim and Rashid [36].

## 4. Materials and Methods

In this section, we describe how to create a feature vector from the projection of cepstral coefficients (CCs) and additional noncepstral metrics extracted from a voice signal. The procedure is inspired by the work of Contreras et al. [8,28], originally proposed for spoofing and dysphonia detection. However, in this study, we introduce three key methodological innovations:

- The use of data augmentation strategies to double the size of the training set through additive noise;
- The integration of noncepstral acoustic features to complement the cepstral representations;
- The systematic experimentation with dimensionality reduction techniques applied to the final feature vector to optimize the compactness and discriminative capacity of the representation.

There are several techniques available for computing CCs. In this work, we consider a diverse set of descriptors, denoted by the set *P*, as defined below:

(2) $$P=\{\Phi_{1},\Phi_{2},\ldots ,\Phi_{n_{P}}\},$$

where each $\Phi_{i}$ is a cepstral coefficient extraction function.

To summarize the information contained in each cepstral matrix and make it suitable for classification tasks, a projection function is applied. While Principal Component Analysis (PCA) was used in the original formulation, we generalize this to include other strategies, such as statistical summarization (mean, standard deviation, skewness), as well as combinations thereof. Let Proj(·) denote a generic projection function or composition of functions (e.g., Proj={PCA,MEAN,STD,SKEW}).

An important goal of this work is to investigate the impact of different dimensionality reduction and projection techniques on the discriminative power of the feature vectors. Unlike previous studies that adopt a single method (usually PCA), we propose a massive and systematic evaluation of a wide range of projection and compression strategies, both at the level of cepstral components and at the final vector level. Our hypothesis is that the compactness and composition of the final vector, including multicepstral and noncepstral elements, plays a crucial role in optimizing performance for voice spoofing detection.

For a voice signal *x*, and a cepstral extraction method ϕ, we compute the matrix of cepstral coefficients $C_{x}^{\phi }$, as well as its first- and second-order derivatives, $\Delta_{x}^{\phi }$ and $\Delta \Delta_{x}^{\phi }$. The feature vector ${\vec{v}}_{x}^{\phi }$ is then defined as

(3) $${\vec{v}}_{x}^{\phi }=\left(\mathrm{Proj}\left(C_{x}^{\phi }\right),\mathrm{Proj}\left(\Delta_{x}^{\phi }\right),\mathrm{Proj}\left(\Delta \Delta_{x}^{\phi }\right)\right),$$

where each Proj(·) maps a matrix $\in R^{n_{\mathrm{ceps}}\times n_{\mathrm{frames}}}$ into a vector in $R^{n_{\mathrm{ceps}}\times n_{\mathrm{proj}}}$, with $n_{\mathrm{proj}}$ depending on the number and type of projection functions used. The fusion vector described in Equation (3) is composed of multiple cepstral representations, including MFCC, CQCC, and others. Although the physical and perceptual principles underlying these representations are not detailed in this work for conciseness, we refer the interested reader to classical works, such as Davis and Mermelstein [37] and Hermansky [38], which provide in-depth modeling and motivation for their use in speech signal processing.

As seen in Section 2, there are noncepstral measures that are extremely useful in speech pattern recognition systems. This is due to the fact that noncepstral measures provide additional information about speech characteristics that may not be captured by cepstral measures. For example, jitter and shimmer reflect amplitude and frequency variations, often associated with vocal anomalies. Other measures, such as mean formant values, intensity, and duration statistics, capture articulation, prosody, and the temporal structure of speech.

Therefore, we propose that the voice signal is also represented by metrics obtained through noncepstral features. Let us consider the set N composed of $n_{N}$ noncepstral metrics capable of representing specific characteristics of the voice signal:

(4) $$N:=\left\{\mu_{1},\mu_{2},\ldots ,\mu_{n_{N}}\right\},$$

where $\mu_{i}:R^{n}\to R^{n_{i}},\forall i\in \left\{1,2,\ldots ,n_{N}\right\}$.

Let us define the feature vector ${\vec{v}}_{x,\mathrm{noncepstral}}$ of a speech signal $x\in R^{n}$ as

(5) $${\vec{v}}_{x,\mathrm{noncepstral}}:=\left(\mu_{1}\left(x\right),\mu_{2}\left(x\right),\ldots ,\mu_{n_{N}}\left(x\right)\right).$$

Finally, to represent the voice signal with both cepstral and noncepstral information, we define the complete feature vector ${\vec{v}}_{x}$ as

(6) $${\vec{v}}_{x}:=\left({\vec{v}}_{x,\mathrm{cepstral}},{\vec{v}}_{x,\mathrm{noncepstral}}\right).$$

The complete process for obtaining the final vector ${\vec{v}}_{x}$ from a signal $x\in R^{n}$ can be described in the following steps:

1. Generation of Data Augmentation: In order to increase the robustness of the feature representations and improve generalization, additive Gaussian noise is applied to each original voice signal. For each sample *x*, a new signal $\tilde{x}$ is generated by $\tilde{x}=x+\epsilon$, where $\epsilon ∼N(0,\sigma^{2})$, effectively doubling the dataset size. The noise variance $\sigma^{2}$ is calibrated to preserve intelligibility while introducing variability.
2. CC Extraction: For each $\Phi_{i}\in P$, compute the matrix of cepstral features: $C_{x}^{\Phi_{i}}:=\Phi_{i}\left(x\right)$.
3. Differential Calculation: Compute the first- and second-order temporal derivatives: $\Delta_{x}^{\Phi_{i}}$ and $\Delta \Delta_{x}^{\Phi_{i}}$.
4. Projection of Features: Apply one or more projection techniques from the candidate set (e.g., PCA, MEAN, STD, SKEW, or combinations). This step is the object of systematic experimentation, and the projected features ${\vec{v}}_{x}^{\Phi_{i}}$ are generated using each strategy in isolation or in combination, as defined in Equation (3).
5. Fusion of Cepstral Representations: Concatenate all intermediate vectors ${\vec{v}}_{x}^{\Phi_{i}}$ into a single feature vector representing all selected cepstral techniques:
  (7) $${\vec{v}}_{x,\mathrm{cepstral}}:=\left({\vec{v}}_{x}^{\phi_{1}},{\vec{v}}_{x}^{\phi_{2}},\ldots ,{\vec{v}}_{x}^{\phi_{n_{P}}}\right).$$
6. Fusion with Noncepstral Features: Concatenate the cepstral feature vector ${\vec{v}}_{x,\mathrm{cepstral}}$ with the noncepstral feature vector ${\vec{v}}_{x,\mathrm{noncepstral}}$, forming the final representation ${\vec{v}}_{x}$, according to Equation (6).
7. Normalization of Data: Standardize ${\vec{v}}_{x}$ using z-score normalization across the training set: $z_{j}=\left(v_{j}-\mu_{j}\right)/\sigma_{j}$, ensuring zero mean and unit variance for each dimension.
8. Final Dimensionality Reduction: Apply a final dimensionality reduction technique to the normalized vector ${\vec{v}}_{x}$, producing a more compact version ${\vec{v}}_{x}^{\mathrm{final}}$. This step is crucial to evaluate the trade-off between dimensionality and model performance and constitutes the core objective of this study.
9. Training of the Classifier: The final reduced vector ${\vec{v}}_{x}^{\mathrm{final}}$ serves as the input for a supervised learning model, trained to discriminate between bona fide and spoofed voice signals. The classifier is fitted using labeled training data $\left({\vec{v}}_{x}^{\mathrm{final}},y_{x}\right)$, where $y_{x}$ denotes the ground truth label. Different classification models, such as Support Vector Machines, Random Forests, and Logistic Regression, are explored under identical conditions to assess their compatibility and performance in conjunction with the dimensionality reduction strategies.

**Figure 1:**
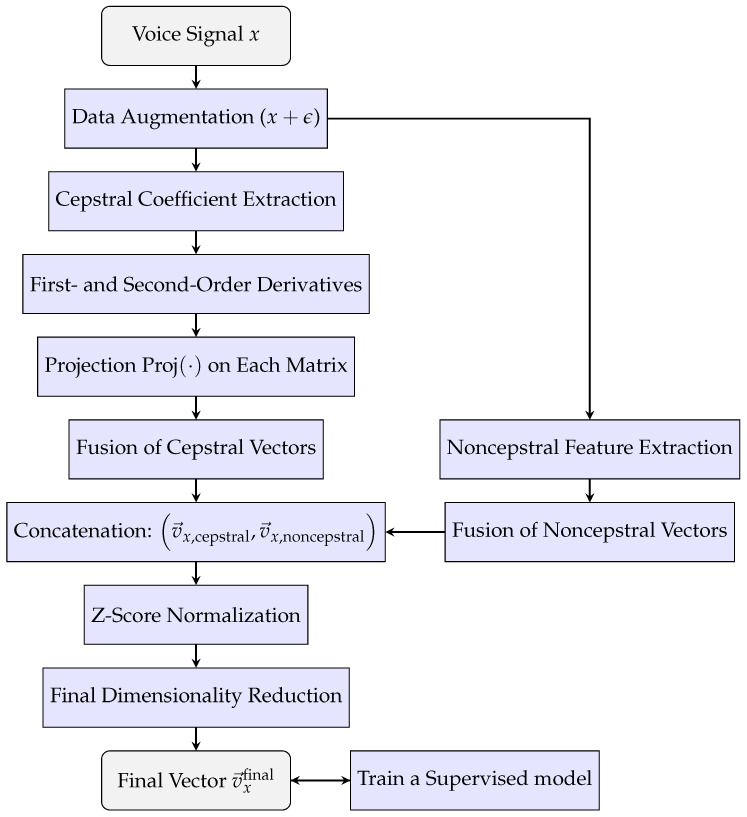
Flowchart of the feature extraction and representation method with multicepstral and noncepstral information.

The overall process is visually summarized in Figure 1.

## 5. Parameters for the Proposed Method and Practical Instances

It is important to emphasize that all the proposed contributions of Contreras et al. [8] were formulated as generalized structures. In this sense, the representation pipeline is parameterized by a set of mapping functions for CC matrix features and a configurable combination of cepstral extraction techniques. Likewise, the framework relies on predefined sets of cepstral and noncepstral features, information aggregation mechanisms, normalization procedures, and data balancing methods.

Because of this flexibility, a concrete instance of the system must be defined in order to execute experimental evaluations. To that end, this work adopts intentionally simplified configurations for each component, with the goal of isolating and assessing the individual contributions of the proposed modeling strategies using accessible and interpretable settings. The following subsection details the specific techniques chosen to instantiate the framework components used in the experimental phase:

- Mapping functions: In the context of this study, the term mapping function refers to a transformation function $m:R^{d\times T}\to R^{d}$ that is applied to each matrix of cepstral features extracted from the voice signal. These matrices typically represent a sequence of *T* frames, where each row corresponds to a cepstral coefficient. The purpose of the mapping is to summarize the temporal evolution of each coefficient into a fixed-size vector. This step is crucial for converting the temporal feature maps into a format compatible with static pattern recognition models. We define this projection step as the function Proj(·) used in Equation (3). For our experiments, we consider four fundamental mapping functions, defined as column-wise operations: (i) projection by PCA ($m_{P}CA$), which reduces the temporal dimension via orthogonal transformation; (ii) column-wise mean (MEAN), which summarizes the central tendency over time; (iii) column-wise standard deviation (STD), capturing temporal variability; and (iv) column-wise skewness (SKEW), reflecting asymmetry in the temporal distribution. These functions may be applied individually or combined in sequence to compose a richer projection strategy.
  In line with the multi-projection concept proposed in this work, we evaluate the sets of mapping functions listed in Table 2. Each configuration corresponds to a different strategy for projecting cepstral matrices into compact feature vectors. The goal is to compare the discriminative potential of different projections and their combinations in the context of voice spoofing detection.
- Noncepstral features: To enrich the representation of the voice signal beyond cepstral information, we incorporate a comprehensive set of noncepstral measures, denoted by N. These features capture complementary acoustic, prosodic, and articulatory cues that may highlight inconsistencies or artifacts introduced by spoofing attacks. Based on the prior literature [39,40,41,42,43,44,45,46], we consider the following metrics:
  – Fundamental frequency statistics: Mean and standard deviation of $F_{0}$, reflecting pitch dynamics over the utterance.
  – Harmonics-to-noise ratio (HNR): Indicates the ratio of periodic (harmonic) to aperiodic (noise) energy in the signal, often altered by synthesis or replay artifacts.
  – Jitter-based features:
    * Local jitter: Measures cycle-to-cycle variability in the pitch period.
    * Local absolute jitter: Captures absolute deviations in pitch period durations.
    * RAP jitter: Relative average perturbation across consecutive cycles.
    * Jitter PCA projection: Single projection summarizing all jitter-based features via PCA.
  – Shimmer-based features:
    * Local dB shimmer: Frame-level amplitude variation in dB.
    * APQ3/APQ5/APQ11 shimmer: Amplitude perturbation quotients calculated over 3, 5, and 11 adjacent cycles, respectively.
    * DDA shimmer: Mean absolute difference in amplitude across voice cycles.
    * Shimmer PCA projection: Single projection summarizing shimmer-related features.
  – Formant-based measures:
    * Mean and median of $F_{1}$–$F_{4}$: Descriptive statistics for the four primary formant frequencies.
    * Formant dispersion: One-third of the distance between $F_{4}$ and $F_{1}$ medians.
    * Arithmetic mean of formants: Mean of the median values of $F_{1}$–$F_{4}$.
    * Formant position: Standardized mean of $F_{1}$–$F_{4}$ medians.
    * Formant spacing (ΔF): Minimum spacing between adjacent formants, estimated via linear regression.
    * VTL based on ΔF: Virtual Tract Length calculated from ΔF spacing.
    * Mean formant frequency (MFF): Fourth root of the product of the medians of $F_{1}$–$F_{4}$.
    * Fitch virtual tract length (FVTL): Estimate of vocal tract length derived from spectral modeling.
- Cepstral coefficient extraction techniques: In this study, we adopt the same diverse set of signal processing methods for extracting cepstral representations as Contreras et al. [8], which are widely used in speaker and spoofing detection tasks. The techniques considered include the following: Constant-Q Cepstral Coefficients (CQCCs), Mel-Frequency Cepstral Coefficients (MFCCs), their inverted variant (iMFCC), Linear Frequency Cepstral Coefficients (LFCCs) [20], Gammatone-based features (GFCCs) [47], Bark-scaled cepstral representations (BFCCs) [48], Linear Predictive Cepstral Coefficients (LPCCs) [49], and Normalized Gammachirp Cepstral Coefficients (NGCCs) [50]. For each technique, the extraction process generates a sequence of feature vectors composed of 20 static coefficients ($n_{\mathrm{ceps}}=20$), along with their respective first and second-order temporal derivatives (Δ and ΔΔ), resulting in a total of 60 dimensions per frame. All features undergo Cepstral Mean and Variance Normalization (CMVN), which standardizes the distribution of each coefficient across frames to zero mean and unit variance, reducing channel and session variability. These cepstral representations are evaluated both individually and in predefined combinations, as organized in Table 3. The goal is to investigate how different spectral scales and filterbank structures influence the discriminative power of the final feature vector when used in conjunction with projection and fusion strategies tailored for voice spoofing detection.
  Due to text space limitations, it was not possible to consider all existing combinations of the eight CC extraction techniques. However, the most important combinations were analyzed to evaluate the performance of the developed material. We consider versions of $P_{1}$ to $P_{8}$, in which each representation of P has only one element. These versions allowed evaluation of how much the framework is enhancing the capacity of the techniques to detect dysphonia in voice signals. In addition, the other versions of CC extraction techniques should serve to confirm the ability of the proposed material to represent different features in the sound sample, which should contribute to improving its ability to detect dysphonia in these samples.
- Normalization: To assess the impact of feature scaling on the final representation ${\vec{v}}_{x}$, we consider two alternative strategies: standard normalization and the absence of normalization. In the first case, each feature is standardized to have zero mean and unit variance across the training set, a procedure commonly used to ensure uniform scale and numerical stability. In the second case, no transformation is applied to the original values, preserving the raw scale of the features. Mathematically, the standardized vector $NORM_{S}TD\left(y\right)$ is given by
  $$NORM_{S}TD\left(y\right):=\left(\frac{y_{1}-\mu_{1}}{\sigma_{1}},\ldots ,\frac{y_{m}-\mu_{m}}{\sigma_{m}}\right),$$
  where $\mu_{i}$ and $\sigma_{i}$ denote the mean and standard deviation of the *i*th feature across the training set. The unnormalized version is obtained by applying the identity function:
  $$NORM_{U}nS\left(y\right):=y.$$
  These two strategies are compared experimentally in order to evaluate whether scaling influences the performance of spoofing detection when applied after fusion and before dimensionality reduction.
- Dimensionality reduction: As a central contribution of this work, we investigate how different dimensionality reduction strategies impact the discriminative power of the final fused vector ${\vec{v}}_{x}$ in the context of spoofing detection. This reduction is applied after the concatenation and normalization of cepstral and noncepstral features, with the aim of eliminating redundancy, enhancing generalization capability, and improving the computational efficiency of the classification stage.
  A total of eight techniques were considered, covering both projection-based and selection-based approaches:
  – PCA: A classical linear projection method that transforms the original feature space into a set of orthogonal components ordered by their ability to capture data variance. The first components are retained to represent the most informative directions in the data.
  – SVD: Similar in nature to PCA, SVD decomposes the data into singular vectors and values, retaining only the most significant components. Unlike PCA, it does not require centering the data and can be applied directly to sparse or high-dimensional matrices.
  – ANOVA F-value selection: A univariate statistical method that selects features by computing the ratio of variance between classes to the variance within classes. Features with the highest discriminative power, as measured by the F-statistic, are selected.
  – Mutual Information (MI) ranking: This technique quantifies the amount of information shared between each feature and the target class. Features are ranked based on their Mutual Information scores, and the most informative ones are retained.
  – Recursive Feature Elimination (RFE): A wrapper method that iteratively trains a predictive model and removes the least important features at each step. This process continues until a desired number of features is retained, prioritizing those most influential to the model’s performance.
  – LASSO-Based selection: A regularization-based approach that performs both feature selection and regression. By applying an $\ell_{1}$ penalty during model training, LASSO forces some coefficients to zero, effectively eliminating less relevant features.
  – Random Forest importance: An ensemble-based selection strategy that evaluates feature relevance based on the contribution of each feature to the accuracy of a collection of decision trees. Features with higher cumulative importance scores are selected.
  – Permutation Importance: A model-agnostic method that estimates the importance of each feature by measuring the drop in predictive performance when the feature values are randomly shuffled. Only features whose permutation leads to a significant degradation in performance are retained.
  All techniques are applied independently to the complete normalized vector ${\vec{v}}_{x}$, producing a compressed version ${\vec{v}}_{x}^{\mathrm{final}}$ that is used as the input to the classifier. By comparing these methods across different reduction levels, this study seeks to understand how representation compactness influences the robustness and effectiveness of spoofing detection systems.
  Each dimensionality reduction strategy presents distinct advantages and limitations that may influence spoofing detection performance in different ways. Projection-based methods, such as PCA and SVD, are particularly efficient in capturing global variance and projecting the data into a dense, lower-dimensional subspace. Their main advantages include simplicity, speed, and the ability to decorrelate features; however, they are linear techniques and may not fully exploit complex nonlinear relationships present in the feature space. Selection-based methods, on the other hand, offer greater flexibility and often produce more interpretable models. ANOVA and MI rely on univariate statistical associations with the target variable, which makes them fast and model-agnostic, but are potentially limited in capturing multivariate interactions. Wrapper methods, like RFE and Permutation Importance, are more powerful in accounting for such interactions but tend to be computationally expensive and sensitive to the choice of the underlying model. Regularization-based selection via LASSO introduces sparsity and robustness to overfitting, yet may underperform when features are highly correlated. Random Forest importance benefits from ensemble-based robustness but can introduce bias towards features with more variability or cardinality.
  By exploring this diverse set of DR techniques, the goal is to identify which methods are more compatible with the fused cepstral and noncepstral representations used in this work, and how their intrinsic properties affect generalization, efficiency, and detection capability. For readers interested in deeper technical insights into each method, we recommend consulting dedicated reviews, such as those by Jia et al. [51], Guyon and Elisseeff [52], and Van Der Maaten et al. [53].
- Classifier: For the classification stage, we adopt a Support Vector Machine (SVM) model using a Gaussian kernel, also known as the Radial Basis Function (RBF) kernel [54]. This choice reflects its wide adoption in speaker verification and spoofing detection tasks due to its ability to model nonlinear decision boundaries effectively. The classifier is trained with class probability estimation enabled, which allows posterior probability scores to be extracted for the computation of evaluation metrics, such as the EER. To improve training stability and mitigate scale sensitivity, the input features are optionally standardized prior to model fitting, using zero mean and unit variance. Additionally, a class-weight adjustment mechanism is applied to reduce sensitivity to class imbalance, ensuring robustness even in experimental configurations that do not incorporate oversampling strategies.

## 6. Results and Experiments

In this section, we present the experimental evaluation designed to assess the performance of the proposed method described in Section 4. To this end, we adopt a benchmark protocol described in detail in Section 6.1, which is widely recognized in the field of voice-based spoofing detection. The experiments aim to analyze the effectiveness of the proposed approach across the various configurations discussed in Section 5, including different combinations of features, projections, normalizations, and reduction techniques. As part of this evaluation, we conduct an in-depth internal analysis of the contribution, exploring how different factors influence performance. This includes the assessment of multiple cepstral representations and their projection strategies, the inclusion or omission of noncepstral features, the impact of applying or skipping normalization, and, most critically, the effect of the dimensionality reduction techniques incorporated into the final feature representation. These experiments are designed to identify which configurations yield the most compact and discriminative representations for spoofing detection.

To compare the effectiveness of the different configurations, we focus on two key evaluation metrics. The first is the accuracy (ACC), which measures the overall proportion of correctly classified instances. The second and most important metric is the Equal Error Rate (EER), which corresponds to the operating point at which the false acceptance rate (FAR) equals the false rejection rate (FRR). This metric is particularly relevant in biometric security contexts, as it reflects the trade-off between rejecting legitimate users and accepting spoofed samples. The EER is computed by analyzing the Receiver Operating Characteristic (ROC) curve and identifying the threshold τ at which the false positive rate (FPR) and the false negative rate (FNR) are equal or as close as possible. In practice, it is estimated by finding the value of the FPR for which the absolute difference between the FPR and FNR is minimized:

(8) $$\mathrm{EER}\approx \mathrm{FPR}\left(\tau^{*}\right)\;\mathrm{where}\;\tau^{*}=\arg\min_{\tau }\left|\mathrm{FPR}(\tau )-\mathrm{FNR}(\tau )\right|.$$

Finally, once the internal analysis is complete, the best-performing configurations will be contrasted with state-of-the-art methods reported in the literature, allowing us to contextualize the effectiveness of our approach and highlight its practical relevance within the spoofing detection domain. All experiments were implemented in the Python version 3.13.5 (https://www.python.org/, accessed on 31 July 2025), using libraries such as Scikit-learn [55], Spafe [56], and Parselmouth [57], and executed on a personal workstation equipped with 8 GB of RAM and an Intel(R) Core(TM) i5-4460 processor running at 3.20 GHz.

### 6.1. Dataset

The dataset adopted for the experiments is the second edition of ASVspoof 2017 [58], which comprises a diverse collection of genuine and spoofed utterances. The genuine recordings originate from the RedDots corpus, captured via Android-based mobile devices and composed of ten predefined secret phrases. The spoofed samples are generated by replaying these original utterances through various playback and recording setups, simulating realistic spoofing attacks under different acoustic conditions. This corpus is organized into three standard partitions, training, development, and evaluation, each reflecting the inherent variability introduced by 177 distinct sessions and 61 different recording configurations. Table 4 summarizes the main characteristics of the dataset and presents the distribution of bona fide and spoofed samples across the three subsets.

### 6.2. Internal Analysis

As described in Section 5, the proposed method supports a wide range of configurable parameters, enabling the construction of multiple experimental instances. In this study, we systematically evaluated thousands of combinations resulting from variations in the feature extraction techniques, projection strategies, inclusion or not of noncepstral features, normalization approaches, and the final dimensionality reduction method. These variations generated a large volume of performance observations over the selected voice spoofing benchmark, which allowed us to explore the behavior of the system under different design choices. In this section, we summarize the key findings from this extensive evaluation process. We highlight the best-performing configurations according to the metrics considered, primarily the EER, and secondarily, the ACC, and provide a structured interpretation of the phenomena observed. The aim is not only to identify effective parameter combinations but also to characterize the impact of each methodological component on the spoofing detection capability of the proposed framework.

Table 5 presents a summary of the best results obtained in terms of accuracy and EER, grouped by set (development or evaluation), use or non-use of noncepstral features, and whether the feature vectors were normalized. For each scenario, we report the maximum accuracy and the minimum EER obtained among all tested configurations. This grouping enables a general understanding of how normalization and the inclusion of noncepstral features affect performance under each condition. The results reveal that the impact of normalization and the inclusion of noncepstral features varies significantly between the development and evaluation sets. In the Dev set, the best performance, both in terms of lowest EER (10.32%) and highest accuracy (87.57%), was achieved without normalization and using only cepstral features, suggesting that the unaltered representation preserves discriminative patterns more effectively in this subset. Conversely, introducing noncepstral features or applying normalization led to a consistent drop in performance, indicating potential overfitting or noise sensitivity. However, in the Eval set, the inclusion of noncepstral features proved beneficial, yielding the lowest EER (10.64%) and highest accuracy (85.58%), regardless of normalization. This suggests that noncepstral features contribute positively to generalization, especially under unseen conditions. Normalization, in turn, had a negligible effect on Eval results, as performance remained unchanged whether it was applied or not. Overall, these findings highlight that while raw cepstral-only representations work best in development, richer representations incorporating noncepstral cues are more robust in evaluation, and that normalization offers limited advantage in either case.

**Figure 2:**
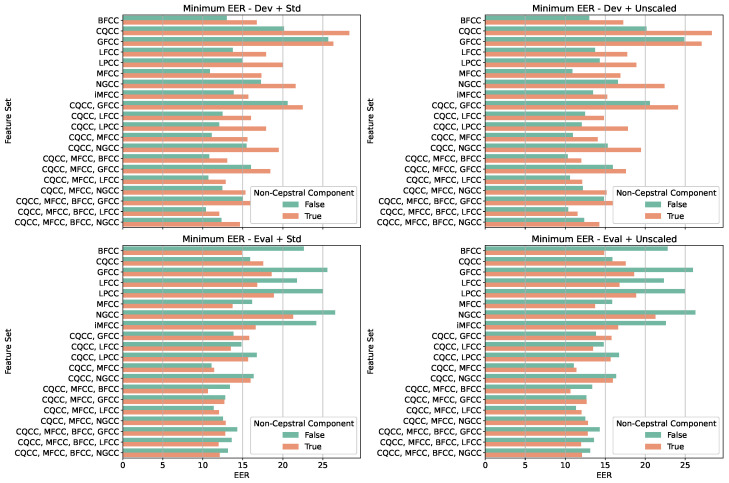
Minimum EERs obtained for each analyzed feature set, organized according to four distinct experimental scenarios. The configuration considers the type of evaluation set used (development or evaluation) and whether data normalization was applied. Bar colors indicate the presence or absence of noncepstral components.

Figure 2 summarizes the best EER values obtained for each combination of cepstral feature sets, grouped by scenario and by the presence or absence of noncepstral features. The four scenarios evaluated correspond to the development and evaluation subsets, each tested with and without normalization.

In the *Dev + Unscaled* condition, the best performances are consistently observed for configurations using only cepstral features, with lower EERs concentrated around techniques such as MFCC, CQCC, and LFCC. This reinforces earlier findings that simpler, unnormalized representations yield better discriminative performance in controlled training environments. In contrast, for the evaluation subset both with and without normalization, the addition of noncepstral features results in a noticeable reduction in EER, suggesting that these features contribute positively to generalization, particularly when the model is exposed to unseen conditions or recording variations. The impact of normalization itself appears to be negligible, as similar EER values are observed across normalized and unnormalized variants within each group. Furthermore, it is worth noting that more complex combinations of cepstral techniques, those involving three or four methods, do not necessarily produce the best results. In many cases, leaner configurations surpass the more elaborate ones, indicating that adding redundant information may not improve the model’s discriminative capacity. Overall, this visualization supports the interpretation that cepstral-only, unnormalized representations are most effective during training, while the integration of noncepstral features enhances robustness during evaluation, with minimal influence from normalization. These findings highlight the importance of evaluating models across both Dev and Eval subsets to ensure the generalizability of observed patterns.

**Figure 3:**
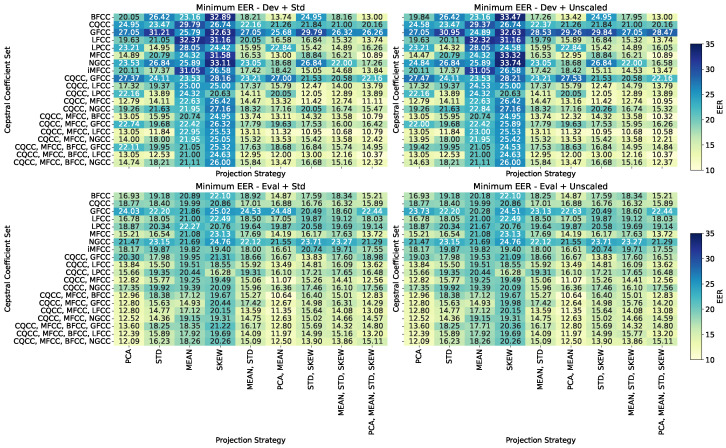
Heatmaps showing the minimum EER values for each combination of projection strategy and dimensionality reduction method. Each subplot corresponds to a different evaluation scenario. This figure enables the identification of the most effective method combinations within each experimental condition.

Rather than enumerating each individual configuration, we focus on describing the most salient and generalizable patterns observed across the heatmaps, to ensure conciseness without compromising analytical depth. In this way, Figure 3 provides a detailed view of the minimum EER values observed for each combination of cepstral coefficient sets and projection strategies, across four experimental conditions: development and evaluation subsets, with and without normalization. The heatmaps reveal consistent patterns regarding the influence of both feature composition and matrix summarization techniques.

Figure 3 reveals multiple insights into the interactions between projection strategies and feature representations. In the *Dev + Unscaled* scenario, lower EER values concentrate around traditional cepstral techniques, such as MFCC and CQCC, when combined with elementary projections, like MEAN or STD. This reinforces previous findings suggesting that, in training-like environments, simpler representations without normalization tend to preserve the discriminative structure of the data more effectively. However, as we move to the *Eval* scenarios, particularly *Eval + Norm*, a shift becomes evident: richer combinations, especially those involving multi-projection strategies (e.g., MEAN + STD, or PCA + MEAN), start to outperform simpler ones, particularly when applied over broader cepstral sets. Notably, while PCA alone is not dominant, its combination with other projections yields competitive results in Eval. Additionally, hybrid cepstral sets like “CQCC, MFCC” or “CQCC, LFCC” show better generalization when coupled with multi-projection. The impact of normalization, once again, appears limited—only slightly modulating performance in some cases, but not altering the global ranking of combinations. These findings suggest that while raw, minimal representations are ideal for in-domain optimization (Dev), enhanced projections and composite cepstral sets offer robustness in evaluation contexts with greater variability.

Table 6 presents a comparative summary of eight dimensionality reduction and feature selection strategies applied to the final feature vector, including both projection-based (e.g., PCA, SVD) and selection-based methods (e.g., LASSO, RFE, RF). The metrics reported are the mean, standard deviation, and minimum EER (%) observed across all evaluated configurations under four experimental conditions: Dev and Eval subsets, with and without standard normalization.

Across all scenarios, the best results are consistently associated with the use of LASSO regularization, which achieves the lowest average EER in both Dev and Eval, regardless of normalization. It is also notable that LASSO achieves the lowest standard deviation, suggesting high stability. PCA and RFE also demonstrate competitive performance, particularly in the Unscaled Dev set, where PCA reaches the absolute minimum EER (10.32%). In contrast, techniques based on univariate ranking, ANOVA and Mutual Information, present the highest mean EERs and lowest minima, indicating poorer overall effectiveness for the task. Interestingly, PIS and RF achieve strong minimum EERs close to the best configurations (e.g., 10.53%, 10.58%), but with significantly higher variances, suggesting less consistency across setups. These results reinforce that embedded methods, such as LASSO and RFE, are better suited to high-dimensional, mixed-feature scenarios, such as the one proposed in this work. Additionally, the overall similarity between normalized and unnormalized variants confirms that normalization has a limited role at this late stage of the pipeline, aligning with earlier findings.

**Figure 4:**
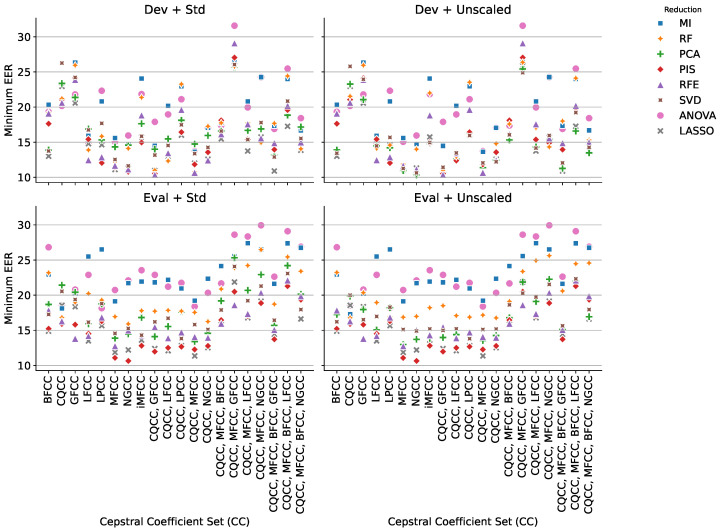
EER achieved for each set of CC, considering different dimensionality reduction techniques across four experimental scenarios. Each subplot corresponds to a specific configuration of evaluation set and data normalization. Colors and shapes identify the reduction technique used. The plot highlights the influence of each method under varying conditions, allowing comparison of robustness and consistency.

Figure 4 illustrates the interaction between cepstral feature sets and dimensionality reduction strategies in terms of the minimum EER achieved across four evaluation scenarios. Each subplot corresponds to a distinct configuration: development and evaluation sets, with and without normalization. The markers represent the reduction techniques, with consistent shapes and colors across all subplots.

Figure 4 enables a detailed comparison of how each dimensionality reduction technique performs across different cepstral coefficient sets and scenarios. A visual inspection reveals that ANOVA-based selection (represented by pink circles) tends to result in the highest EERs, regardless of the cepstral set or scenario, indicating that univariate selection techniques are not well suited to this problem. On the other hand, RFE (likely shown as purple triangles) performs competitively in the development scenarios, often occupying the lower EER band, particularly when paired with MFCC, CQCC, or their combinations. This suggests that recursive elimination is effective for tuning performance under training conditions. In contrast, PIS (shown as red diamonds) stands out in the evaluation sets, repeatedly achieving low EER values, especially when combined with richer cepstral sets. This implies that PIS provides better generalization in more diverse testing environments, likely due to its model-aware selection of features. Additionally, LASSO-based methods (gray × markers) appear stable across all settings, maintaining relatively low EER with reduced variance, corroborating prior observations from the tabular summary. The facet structure makes it clear that while some reduction strategies excel in Dev, their advantages do not necessarily carry over to Eval. This visualization reinforces the need to assess reduction techniques not only by their training performance but also by their stability and generalization under evaluation conditions.

**Figure 5:**
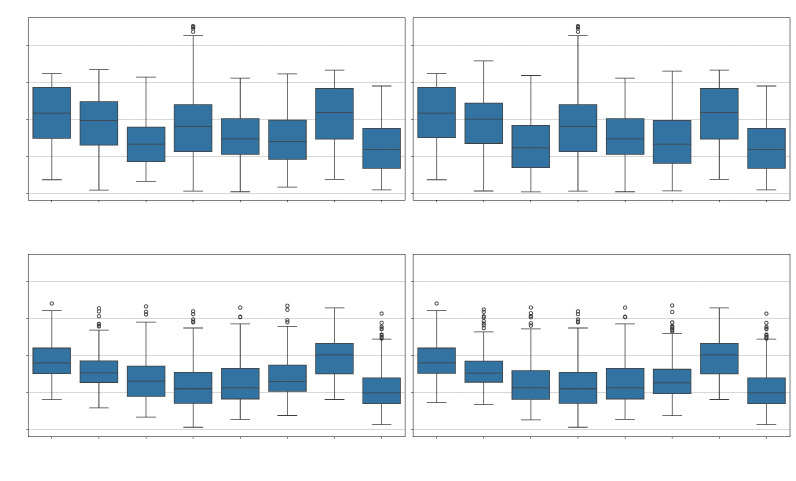
EER for each dimensionality reduction technique, analyzed separately by experimental scenario. Each boxplot summarizes the central tendency, dispersion, and outliers of the EER values across different combinations of projection and feature sets.

Figure 5 presents boxplots illustrating the distribution of EER values achieved by each dimensionality reduction technique across the four experimental scenarios. These visualizations highlight not only central tendency (medians) but also the variability and presence of outliers for each method under each condition.

In both development scenarios, LASSO exhibits the most compact distribution, with consistently low medians and reduced variance, confirming its previously observed stability. RFE also shows competitive median values in Dev, albeit with slightly wider dispersion. Interestingly, PCA and SVD, despite being classical projection-based methods, show moderate performance with larger variability, indicating sensitivity to the feature configuration and possibly limited robustness. On the other hand, evaluation scenarios reveal a shift in behavior. PIS presents one of the lowest median EERs in Eval, particularly in the unnormalized condition, while still maintaining a relatively tight interquartile range. This supports the earlier finding that PIS generalizes well to unseen data. LASSO remains among the best also in Eval, further validating its consistency across different settings. Techniques such as ANOVA and Mutual Information consistently exhibit both higher medians and a wider spread across all scenarios, reinforcing their lower effectiveness and greater instability in this application. Notably, Random Forest-based selection, while competitive in some isolated instances, shows broad variability in EER performance, making it less predictable as a general strategy. Overall, the boxplots corroborate previous quantitative results and emphasize that LASSO and PIS are the most balanced options, offering both competitive accuracy and stable behavior across evaluation conditions. Meanwhile, univariate filters (ANOVA, MI) remain the least reliable choices in the context of dimensionality reduction for spoofing detection.

### 6.3. State-of-the-Art Comparison

To assess whether the multicepstral feature representations adopted in the proposed framework are competitive with state-of-the-art solutions, we conduct a comparative analysis between the best results achieved by our approach and those reported in recent publications that evaluate models on the “ASVSpoof 2017 v2.0” dataset. Table 7 summarizes the lowest EER values reported by leading methods in the literature, considering both the development set, when applicable, and the evaluation set of the benchmark. Additionally, the final column presents the average of these two metrics, providing an aggregated measure of performance for comparison purposes.

As shown in Table 7, the proposed method achieves competitive results when compared to a broad spectrum of recent state-of-the-art approaches applied to the ASVSpoof 2017 v2.0 corpus. In terms of EER, our method reports values of 10.32% on the development set and 10.64% on the evaluation set, resulting in a mean of 10.48%. This average places the method among the best-performing techniques overall, especially considering that many alternative methods rely on deep models, handcrafted combinations of cepstral variants, or ensemble classifiers.

While a few approaches, such as those based on feature-rich ensembles (e.g., CQCC + CFCCIF + ESA) or tuned cepstral hybrids (e.g., CQCC + QCFCCIF-ESA), report lower average EERs, it is important to highlight that several of them exhibit high variance between development and evaluation results, suggesting a potential lack of generalization. For instance, some methods that perform exceptionally well on the development set (e.g., OMSp-HASC-Canny) present significant degradation on the evaluation data. In contrast, the proposed method maintains a balanced performance across both partitions, with a negligible gap between Dev and Eval.

This result supports the robustness of the multicepstral and projection-based strategy adopted in this study, indicating that the fusion of cepstral and noncepstral features, combined with a systematic dimensionality reduction pipeline, can achieve performance on par with more complex architectures while offering transparency and flexibility for further adaptation to different spoofing scenarios.

## 7. Conclusions

Ensuring robust spoofing detection in voice biometric systems is a critical step toward strengthening the security of authentication frameworks. In this study, we proposed and systematically evaluated a configurable and extensible framework that integrates multicepstral representations, noncepstral acoustic features, and a diverse set of dimensionality reduction techniques. The objective was to analyze the impact of these components on the EER under different normalization and evaluation conditions using the ASVSpoof 2017 v2.0 benchmark.

Our findings reveal that no single combination of techniques universally outperforms others; rather, performance is scenario-dependent. Nonetheless, some trends were evident: LASSO-based dimensionality reduction consistently demonstrated strong generalization with low EER and low variance, whereas univariate filters, like ANOVA and Mutual Information, underperformed across all cases. PIS emerged as particularly effective in evaluation subsets, suggesting superior adaptability to unseen data. Moreover, RFE yielded competitive results in development settings. These outcomes emphasize that the fusion of multicepstral and noncepstral features, when combined with appropriate projection and reduction strategies, can match or outperform more complex deep learning pipelines with greater transparency and flexibility.

The proposed method achieved one of the lowest average EERs among the recent literature, reinforcing its relevance in practical biometric security applications. The framework also allows easy adaptation and experimentation, enabling the investigation of new combinations without reengineering the entire pipeline.

Building upon these results, several research directions emerge. First, future work may explore the inclusion of additional noncepstral features, such as phase-based or articulatory-based representations, which may offer complementary discriminative power. Second, given the promising results from PIS and LASSO, it would be valuable to evaluate hybrid reduction strategies that combine model-aware importance and regularization constraints.

Another promising path involves the integration of sound texture representations derived from spectrogram analysis, inspired by texture-based spoofing detection in other biometric modalities, such as fingerprints. Techniques such as Local Binary Patterns or Gabor filters applied over time-frequency representations may encode perceptual distortions caused by replay attacks. Additionally, since this study focused on shallow and interpretable models, future work may involve comparing these handcrafted pipelines against explainable variants of deep models, assessing the trade-off between transparency and raw performance.

Finally, considering the effectiveness of genetic-based optimization in prior works, we intend to investigate advanced metaheuristic techniques, such as massive local search operators and hybrid evolutionary schemes, aiming to refine the dimensionality reduction phase and further enhance detection performance in complex and adversarial conditions.

## Figures and Tables

**Table 1:** Comparative summary of related works on voice spoofing detection.

Authors	Technique/Approach	Dataset	Best EER	Main Contributions
Patel and Patil [19]	GMM with CFCC + IF	ASVspoof 2015	0.4079% (known)	Simulation of human auditory processing using cochlear filters and instantaneous frequency
Sahidullah et al. [20]	Comparison of 19 feature sets + GMM/SVM	ASVspoof 2015	0.07% (known)	Comprehensive evaluation of spectral, phase, and long-term features
Todisco et al. [21]	Constant-Q Transform	ASVspoof 2015	0.048% (known)	Adaptation of music signal processing technique for spoofing detection
Yang and Das [22]	CQT + low-frequency frame normalization + SVM/ResNet	ASVspoof 2017	10.31%	Enhanced robustness to noise and environmental variability
Hanilçi [23]	i-vectors + GMM	ASVspoof 2015	2.39%	EER reduction by combining traditional modeling with i-vectors
Qian et al. [24]	DNN, AE, LSTM-RNN	ASVspoof 2015	≈0% (known)	Comparison of five deep neural architectures
Huang and Pun [25]	DenseNet + LSTM with MFCC + CQCC	ASVspoof 2017	7.34%	Fusion of two deep networks with multicepstral features
Gomez-Alanis et al. [26]	Gated Recurrent CNN	ASVspoof 2015/2017/2019	0%	Improved robustness to real-world noise
Himawan et al. [30]	AlexNet (transfer learning) + SVM	ASVspoof 2017	11.21%	Use of pre-trained CNN to mitigate overfitting and extract embeddings
Contreras et al. [8]	Fusion of MFCC, CQCC, BFCC, and LFCC with multiple projections	ASVspoof 2017	15.02%	Introduction of multicepstral-enriched vector mappings
Contreras et al. [29]	GA, PSO, GWO for selection in multicepstral spaces	ASVspoof 2017	17.42%	Metaheuristic optimization in multicepstral representations
Contreras et al. [12]	SVD, AE, GA for dimensionality reduction	ASVspoof 2017	13.41%	Dimensionality reduction in multicepstral spaces with EER improvement

**Table 2:** Evaluated sets of mapping functions M used for projecting cepstral matrices.

ID	Projection Strategies
Proj_1	{PCA}
Proj_2	{MEAN}
Proj_3	{STD}
Proj_4	{SKEW}
Proj_5	{PCA, MEAN}
Proj_6	{MEAN, STD}
Proj_7	{STD, SKEW}
Proj_8	{MEAN, STD, SKEW}
Proj_9	{PCA, MEAN, STD, SKEW}

**Table 3:** Considered configurations for the sets of CC extraction techniques used in the experiments.

Set	CC Extraction Technique(s)
$P_{1}$	MFCC
$P_{2}$	CQCC
$P_{3}$	iMFCC
$P_{4}$	BFCC
$P_{5}$	LFCC
$P_{6}$	LPCC
$P_{7}$	GFCC
$P_{8}$	NGCC
$P_{9}$	CQCC, MFCC
$P_{10}$	CQCC, LFCC
$P_{11}$	CQCC, LPCC
$P_{12}$	CQCC, GFCC
$P_{13}$	CQCC, NGCC
$P_{14}$	CQCC, MFCC, LFCC
$P_{15}$	CQCC, MFCC, BFCC
$P_{16}$	CQCC, MFCC, GFCC
$P_{17}$	CQCC, MFCC, NGCC
$P_{18}$	CQCC, MFCC, BFCC, NGCC
$P_{19}$	CQCC, MFCC, BFCC, LFCC
$P_{20}$	CQCC, MFCC, BFCC, GFCC

**Table 4:** Details of the ASVSpoof 2017 v2.0 benchmark.

Subset	Speakers	Sessions	Settings	Genuine	Spoofing
Training	10	6	3	1507	1507
Development	8	10	10	760	950
Evaluation	24	161	57	1298	12,008
Total	42	177	70	3565	14,465

**Table 5:** Best accuracy (max) and lowest EER (min) by evaluation configuration.

Set	Normalized	Noncepstral	Accuracy (max)	EER (min)
Dev	No	No	87.57	**10.32**
	Yes	No	86.58	10.37
	No	Yes	80.56	11.53
	Yes	Yes	79.42	12.05
Eval	No	No	83.31	11.07
	Yes	No	83.31	11.07
	No	Yes	85.58	**10.64**
	Yes	Yes	85.58	**10.64**

**Table 6:** Comparison of dimensionality reduction and feature selection techniques based on average, minimum, and standard deviation of EER (%) across all configurations.

Scenario	Technique	EER Mean	Standard Deviation	EER Min
Dev + Std	LASSO	22.31	6.39	10.89
	PCA	23.67	5.78	13.21
	SVD	24.57	6.66	11.63
	RFE	24.79	6.55	10.37
	PIS	27.99	9.41	10.53
	RF	28.69	7.46	10.79
	MI	31.12	8.18	13.63
	ANOVA	31.27	7.76	13.74
Dev + Unscaled	LASSO	22.31	6.39	10.89
	PCA	22.83	6.76	10.32
	SVD	23.74	7.04	10.63
	RFE	24.80	6.56	10.37
	PIS	27.99	9.41	10.53
	RF	28.63	7.34	10.58
	MI	31.13	8.17	13.63
	ANOVA	31.27	7.76	13.74
Eval + Std	LASSO	21.08	5.94	11.35
	PIS	22.05	6.37	10.64
	RFE	22.79	6.02	12.76
	PCA	23.58	5.90	13.39
	SVD	24.03	5.59	13.82
	RF	25.86	4.67	15.90
	MI	28.84	4.90	18.10
	ANOVA	29.71	5.12	18.15
Eval + Unscaled	LASSO	21.08	5.94	11.35
	PIS	22.05	6.36	10.64
	PCA	22.39	5.98	12.64
	RFE	22.79	6.02	12.76
	SVD	23.49	5.46	13.80
	RF	25.97	4.76	16.76
	MI	28.83	4.91	17.30
	ANOVA	29.71	5.12	18.15

**Table 7:** Comparison of the proposed method with state-of-the-art spoofing detection approaches on the ASVSpoof 2017 v2.0 dataset. The table reports the best EER (%) values obtained for each method on the development and evaluation subsets, along with the average between them.

Algorithm	EER (%)		
	Dev	Eval	Mean
**Proposed Method**	10.32	10.64	10.48
2D-ILRCC [59]	11.90	10.87	11.38
iMFCC/SCMC [60]	4.83	11.49	8.16
OMSp-HASC-Canny [61]	3.52	22.86	13.19
CQCC, MFCC (PCA) [29]	-	17.42	17.42
CQCC, MFCC, LFCC [28]	-	15.02	15.02
LFCC + CQCC + LTAS [62]	8.60	26.10	17.35
SWM-CQCC-DA [63]	-	10.27	10.27
LCNN/smallCNN [64]	7.37	10.70	9.03
CQCC + CFCC + CFCCIF + CFCCIF-ESA/QESA [65]	1.88	10.99	6.43
LFDCC/CDOC [66]	13.03	11.63	12.33
CQCC + QCFCCIF-ESA [67]	2.19	11.00	6.59
CQCC + JS [68]	7.60	10.90	9.25
EMD-HS-RFCC + CQCC + APGDF [69]	4.89	10.84	7.86
ResNet/LCNN [70]	7.85	8.20	8.02
CQCC + LFCC + MFCC + u-vector [71]	6.66	11.99	9.32
TECC [72]	10.80	11.41	11.10
CQCC + AT-IMFCC + AT-MelRP [73]	2.08	10.75	6.41
(LPCC + LFCC)-GMM [74]	5.26	22.65	13.95
CQCCs + AFCCsFAF + ARPDBF [75]	1.72	11.22	6.47
Multicepstral features + ResNet/GMM [76]	7.57	11.64	9.60
CQCC-CNN [77]	7.37	10.70	9.03
TDNN + 2PLDA − CQCC + MFCC [78]	-	11.16	11.16
CQSPIC [79]	-	11.34	11.34
ESA-IACC-IFCC [80]	7.03	10.12	8.57
GMM + CQCC-${\mathrm{MP}}_{\mathrm{aware}}$ DNN + (MFCC + MGDCC) [81]	5.86	24.14	15.00
CQCC (90-D (SDA)) − AWFCC [82]	5.75	10.42	8.08
LFCC [83]	7.02	14.83	10.92
(eCQCC-STSSI)-DA [84]	-	10.07	10.07
CVOC-DA [85]	-	11.46	11.46
SFCC-QCN [86]	8.38	10.11	9.24
Spectrogram-CNN [87]	10.82	16.03	13.42
CQCC + VTECC [88]	5.85	10.94	8.39
CQNCC-D DNN [22]	-	10.31	10.31
CQCC + PNCC [89]	8.73	12.98	10.85
CQCC-CMVN [58]	9.06	13.74	11.40

## Data Availability

The original data presented in this study are openly available in DataShare [90] at https://doi.org/10.7488/ds/2332, accessed on 31 July 2025.
